# Overtime trend of thyroid hormones and thyroid autoimmunity and ovarian reserve: a longitudinal population study with a 12-year follow up

**DOI:** 10.1186/s12902-019-0370-7

**Published:** 2019-05-07

**Authors:** Sara Bahri, Fahimeh Ramezani Tehrani, Atieh Amouzgar, Maryam Rahmati, Maryam Tohidi, Maryam Vasheghani, Fereidoun Azizi

**Affiliations:** 1 Reproductive Endocrinology Research Center, Research Institute for Endocrine Sciences, Shahid Beheshti University of Medical Sciences, Tehran, Iran; 2 Endocrine Research Center, Research Institute for Endocrine Sciences, Shahid Beheshti University of Medical Sciences, Tehran, Iran; 3 Department of Epidemiology and Biostatistics, School of Public Health, Tehran University of Medical Sciences, Tehran, Iran; 4 Prevention of Metabolic Disorders Research Center, Research Institute for Endocrine Sciences, Shahid Beheshti University of Medical Sciences, Tehran, Iran; 5 Chronic Respiratory Diseases Research Center, National Research Institute of Tuberculosis and Lung Diseases (NRITLD), Shahid Beheshti University of Medical Sciences, Tehran, Iran; 6 Endocrine Research Center, Research Institute for Endocrine Sciences, Shahid Beheshti University of Medical Sciences, Tehran, Iran

**Keywords:** Anti-mullerian hormone, Ovarian reserve, Tehran thyroid study (TTS), Thyroid autoimmunity, Thyroid hormones

## Abstract

**Background:**

Ovarian reserve, vital for reproductive function, can be adversely affected by thyroid diseases. Despite alternations of thyroid hormones with ageing, data on interactions between the overtime trend of thyroid functions and ovarian reserve status has rarely been reported. We aimed to examine the overtime trend of thyroid hormones, thyroid peroxidase antibody (TPO Ab) and their associations with ovarian reserve status, identified by levels of age specific anti-mullerian hormone (AMH) in reproductive aged women, who participated in 12-year cohort of Tehran Thyroid Study (TTS).

**Methods:**

Reproductive age women(*n* = 775) without any thyroid disease or ovarian dysfunction were selected from the Tehran Thyroid Study cohort. Participants were divided into four age specific AMH quartiles (Q1-Q4), Q1, the lowest and Q4, the highest. AMH was measured at the initiation of study and thyroid stimulating hormone (TSH), free T4 (FT4), and TPO Ab were measured at baseline and at three follow up visits.

**Results:**

At baseline, there was no statistically significant difference in thyroid hormones between women of the four quartiles, although TPO Ab levels were higher in women of Q1. During the follow ups, FT4 was decreased in all quartiles (*p* < 0.05), whereas TPO Ab increased in Q1 (*p* = 0.02). Odds ratio of overall TPO Ab positivity in women of Q1 was 2.08 fold higher than those in Q4. (OR: 2.08, 95%CI: 1.16, 3.72; *p* = 0.01).

**Conclusion:**

Women with the lowest ovarian reserves had higher levels of TPO Ab, with a positive trend of this antibody overtime in comparison to other quartiles, indicating that this group may be at a higher risk of hypothyroidism over time.

## Background

Ovarian reserve is vital for reproductive function, and women with reduced ovarian follicles are at an increased risk of premature ovarian failure (POF) [1]. The status of ovarian reserve can be precisely measured by anti-Müllerian hormone (AMH), which is secreted by the granulosa cells of ovarian follicles [2]. Among the various factors assumed to be associated with POF [2, 3], thyroid function status and thyroid autoimmunity have in particular, been suggested [4]. Thyroid dysfunction influences the reproductive system directly by affecting oocytes, via thyroid hormone receptors on the surface of these cells [4], and indirectly through increasing prolactin secretion and disruption of GnRH function [5]. In addition it has been shown that thyroid autoimmune disease may be associated with a general autoimmunity, leading to premature ovarian failure; however the main pathophysiology linking ovarian reserves with TPO antibody and thyroid hormones has not been elucidated [6–8]. In a cross sectional study of 5000 Belgian women (mean age: 32.0, SD: 5.5 years), there were no significant association, between ovarian reserve, thyroid hormones and thyroid autoimmunity [9]. Several studies also show an inverse association between ovarian reserves, hypothyroidism and thyroid autoimmunity in humans and animals [10, 11]; however to the best of our knowledge, the interaction between changes in thyroid functions and ovarian reserves status has not yet been reported and it is unclear whether or not women with poor ovarian reserves are at increased risk of occurrence of hypothyroidism.

We aimed to assess the trend of changes in thyroid hormones and TPO Ab among reproductive aged women by various ovarian reserve statuses, identified by age- specific AMH levels in a population based cohort study, the Tehran Thyroid Study, with an average 12 years of follow up.

## Methods

### Study population

We used data from the participants of the Tehran Thyroid Study (TTS) [12], a population-based cohort study being conducted within the framework of the Tehran Lipid and Glucose Study (TLGS) [13]. In the TLGS, 15005 people, aged ≥3 years, living in district No. 13 of Tehran, were selected by a multistage cluster random sampling method; Detailed descriptions of the TLGS have been published elsewhere [14]; of these 4174 individuals (male and female), aged ≥20 were included in the Tehran Thyroid Study.

The TTS aimed at determining the prevalence and natural courses of thyroid disease, and to demonstrate the relationship between thyroid disease and cardiovascular risk factors, ischemic heart disease, and all-cause mortality in the population of Tehran. In the current prospective study, participants were followed every 3 years interval over a 12 year follow up.

Data on smoking status, history of radioiodine exposure, physical activity levels, history of thyroid surgery, and using levothyroxine or anti-thyroid medications were obtained during face to face interviews every 3 years by trained staff. At each follow up, participants were also asked about their reproductive history, including marital status, regularity of menstrual cycle, parity, and the current and previous contraceptive methods used. If their menstrual cycles had ceased, the date of the last cycle was recorded. With exception of serum AMH, all biochemical variables including thyroid hormones, TPO Ab and TSH were measured at each follow-up, every 3 years.

For the purpose of present study, we selected women, aged 20–50 years at baseline who met eligibility criteria, which included: Having regular and predictable menstruation at initiation of study and documented natural fertility; exclusion criteria were: history of endometriosis, Poly Cystic Ovarian Syndrome (PCOS), hysterectomy, oophorectomy or any other kind of ovarian surgery, using hormonal replacement for menopausal symptoms at the initiation of the study or during follow-up; and usage of hormonal contraception for ≥3 months before entering the study. Those with history of thyroidectomy, radio iodine treatment, and those using of levothyroxine, amiodarone, glucocorticoids or anti thyroid dugs were also excluded. Participants were divided into four age specific AMH quartiles (Q1-Q4); (Q1: the lowest and Q4: the highest). Women included had to have complete data and hormonal profiles for all follow-ups. All participants signed written informed consent forms and the study was approved by the ethics committee of the Research Institute for Endocrine Sciences, RIES affiliated, to the Shahid Beheshti University of Medical Sciences. IR ENDOCRINE,REC.1396.416.

### Measurements

All measurements were conducted at baseline and each of the three follow ups. Anthropometric measures including weight and waist circumference (WC), were measured at the umbilicus level using an unstretched tape meter, without any pressure to body surface; Body Mass Index (BMI) was calculated as weight in kilograms (kg) divided by height in squared (m^2^). Systolic blood pressure (SBP) and diastolic blood pressure (DBP) were measured twice using a standard mercury sphygmomanometer, in a seated position after a 15-min rest period, and the mean of these two measurement was considered as the subject’s blood pressure.

Blood samples were taken from the subjects between 7:00 AM and 9:00 AM while sitting, using vacutainer tubes after a 12-h overnight fast. Samples were centrifuged within 30–45 min of collection and stored at -80C. At baseline the serum concentration of anti-Müllerian hormone (AMH) was measured by the two-site enzyme immunoassay (EIA) method; the kit was the Gen II kit (Beckman Coulter, Inc., CA, USA) and intra- and inter-assay CVs were 1.9 and 2.0%, respectively and the reader was Sunrise ELISA reader (Tecan Co. Salzburg, Austria). All AMH measurements were taken simultaneously at the same laboratory. AMH Gen II controls A79766 were used at two levels of concentration to monitor accuracy of the assay. Serum TSH and free T4 concentrations were measured by the electro the chemi luminescence immunoassay (ECLIA) method, using commercial kits on the Cobase e - 411 analyzer (Roche Diagnostics GmbH, Mannheim, Germany). The inter- and intra-assay CVs were 3.3 and 1.3% for free T4 and 4.6 and 1.4% for TSH determination, respectively. Sensitivities of free T4 and TSH measurement were 0.28 pmol/L and 0.005 mU/L, respectively. The reference values for TSH were (0.32–5.06) mU/L and the reference values for T4 were (11.58–19.95) pmol/L. TPOAb was assayed by immune enzymometric assay (IEMA-Monobind, Costa Mesa, CA, USA), using the Sunrise ELISA reader (Tecan Co., Salzburg, Austria); inter- and intra-assay CVs were 4.2 and 3.9%, respectively. The sensitivity of the assay was 0.92 IU/mL. Serum AMH level was measured one time at the beginning of study, while all other biochemical measurements were conducted at baseline and also at each of the three follow ups.

### Definitions

According to normal-based methodology of Altman and Chitty [15] and Royston and Wright [16], age-specific AMH percentiles were estimated based on AMH levels. With AMH centiles, age-specific AMH was calculated, using the exponential–normal 3-parameter model; the cut-off values for age-specific quartiles have previously been described in detail [17]. Individuals were initially placed in one of percentiles and eventually in the quartile) categories of age specific AMH level. Cut off values calculated in Tehran Thyroid Study [18] were used for definition of various thyroid dysfunctions as follows: Hypothyroidism TSH > 5.06 mU/L and FT4 < 11.58 pmol/L; subclinical hypothyroidism TSH > 5.06 mU/L and normal FT4 (11.58–19.95) pmol/L; hyperthyroidism TSH < 0.32 mU/L and FT4 > 19.95 pmol/L and subclinical hyperthyroidism TSH < 0.32 mU/L and normal FT4 [12]; TPO Ab > 35 IU/mL was considered as TPO Ab positivity [18]. Smoking was considered as a binomial variable as: ever smokers: (using ≥1 cigarette per day) and never smokers.

### Statistical analysis

Baseline characteristics are presented as mean (standard deviation) for numerical variables and number (percentage) for the categorical measures. For numerical variables with skewed distribution, median (inter quartile range) was calculated.

Differences in descriptive baseline characteristics of women of various AMH quartiles were explored using analysis of variance. The Kruskal-Wallis test was applied to compare baseline values of variables with skewed distributions. Chi square test was used to compare the prevalence of thyroid disorders in the quartiles of AMH. To analyze the person-years incidence rate of thyroid disorders the following formula was used:

$$ \frac{\mathrm{Number}\ \mathrm{of}\ \mathrm{new}\ \mathrm{events}\ \mathrm{of}\ \mathrm{the}\ \mathrm{condition}\ \left(\mathrm{cases}\right)\ \mathrm{during}\ \mathrm{the}\ \mathrm{study}\ \mathrm{time}}{\mathrm{Sum}\ \mathrm{of}\ \mathrm{person}-\mathrm{time}\ \left(\mathrm{person}\times \mathrm{year}\right)\mathrm{at}\ \mathrm{risk}\ \mathrm{among}\ \mathrm{the}\ \mathrm{study}\ \mathrm{participants}} $$

The hazard ratio, which is derived from the Cox proportional hazards model, provides a statistical test of comparing incidence rates of thyroid disorders in age-specific quartiles of AMH. The hazard ratio is equivalent to the odds that an individual in the group with higher hazard reaches the endpoint first [19]. After excluding those with history of thyroidectomy, radio iodine treatment, and those using of levothyroxine or anti thyroid dugs during the follow ups, the generalized estimation equation (GEE) method was used as an estimation method for marginal, i.e. population-averaged modeling of repeated data [20] to investigate the secular longitudinal trends of TPO Ab, FT4, and TSH. Models for examination of time trends were fitted separately for the first and fourth quartiles of AMH and *P* values for trend were reported in each quartile. This model included the following predictors: Time (follow-up years), AMH quartile status, and an interaction term of these two (follow-up years × AMH quartile status) and was adjusted for BMI, smoking, parity, and education level. This interaction shows how the effect of AMH quartile status on thyroid functions changed overtime.

Statistical analysis was performed, using the software package STATA (version 12; STATA Inc., College station, TX, USA); significance level was set at *P* < 0.05, and CI as 95%.

## Results

**Fig. 1:**
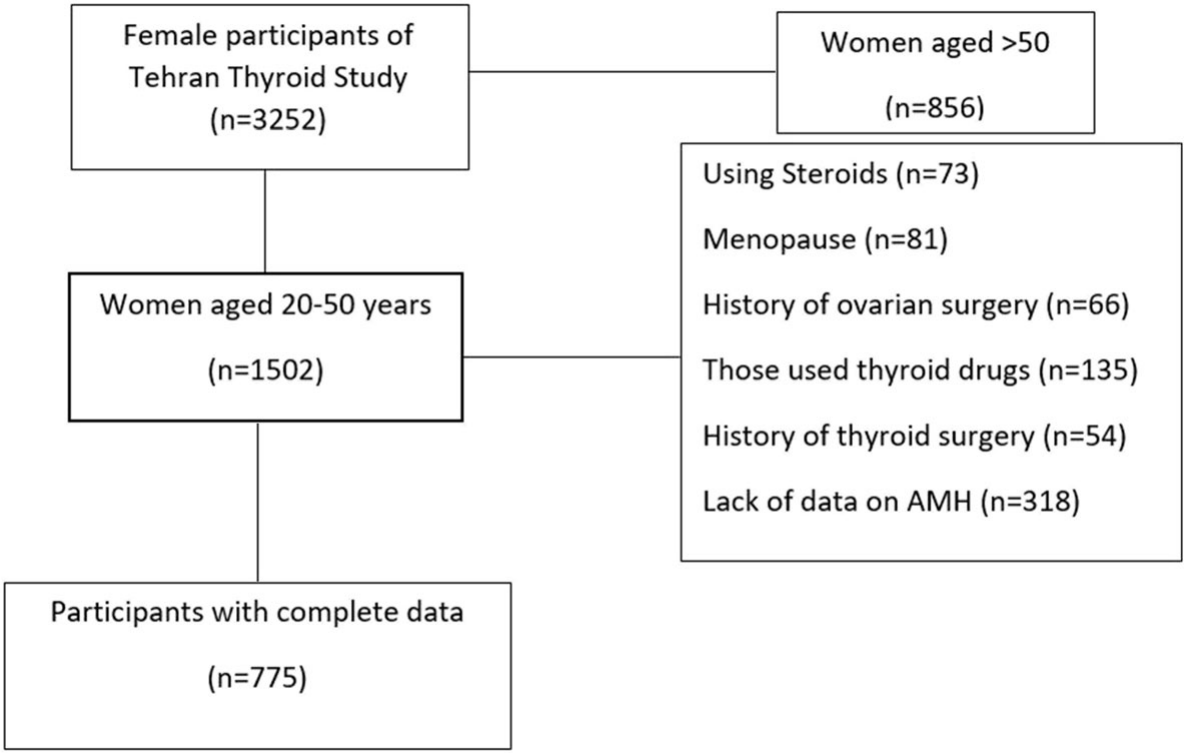
Flow chart of the study

Figure 1 illustrates the study flowchart; of 4174 participants of the Tehran Thyroid Study, there were 1502 women, aged between 20 and 50 years; among these, 775 met our eligibility criteria. At baseline, 203, 181, 201 and 190 subjects were in the first (Q1 or lowest level), (Q2) second, (Q3) third and fourth (Q4 or highest level) quartiles of age-specific AMH, respectively; their baseline characteristics are presented in Table 1. Initially, there was no statistically significant difference in the anthropometric measures, diastolic blood pressures, parity, smoking status, educational level, and thyroid hormone levels among participants of various quartiles. Women in the first quartile had higher levels of TPO Ab; 7.85(3.69, 23.3) IU/ml in comparison with those in the 2nd, 3rd and 4^th^quartiles 5.59 (3.10, 18.66), 5.14 (3.01, 11.35), 6.32 (3.37, 13.89), respectively; *p*-value = 0.02.

**Table 1:** Baseline characteristics of study subjects according to the age-specific anti-Mullerian hormone (AMH) quartiles

Variable	1st quartile *n* = 203	2nd quartile *n* = 181	3rd quartile *n* = 201	4th quartile *n* = 190	*P*- value
Age(years)	38.3 ± 6.7	37.3 ± 6.5	36.4 ± 6.8	37.2 ± 6.7	0.42
BMI (kg/m^2^)	26.8 ± 4.1	26.8 ± 4.4	27.1 ± 4.4	27.4 ± 4.5	0.44
WC (cm)	85.3 ± 9.8	84.7 ± 11.3	85.4 ± 10.8	86.0 ± 11.2	0.70
HC (cm)	104.0 ± 7.9	104.1 ± 8.5	104.3 ± 9.1	104.6 ± 9.0	0.91
WHR	0.816 ± 0.6	0.810 ± 0.7	0.816 ± 0.6	0.820 ± 0.7	0.64
SBP (mm/Hg)	113.3 ± 13.9	108.9 ± 12.9	111.9 ± 14.9	111.4 ± 12.4	0.01ª
DBP (mm/Hg)	75.3 ± 9.92	73.9 ± 9.2	75.3 ± 10.1	75.2 ± 8.9	0.38
Parity (n)	3.3 ± 1.7	3.0 ± 1.4	3.1 ± 1.6	3.0 ± 1.7	0.73
FT4 (pmol/L)	14.67 ± 2.13	23.94 ± 4.50	21.36 ± 2.57	21.11 ± 4.50	0.36
TSH (mu/L)	2.05 (1.03,3.13)	1.86 (1.06,3.72)	1.66 (1.00,2.75)	1.64 (0.92,2.82)	0.26
TPO Ab (IU/ml)	7.85 (3.69,23.3)	5.59 (3.10,18.66)	5.14 (3.01,11.35)	6.32 (3.37,13.89)	0.02ª
Smoking Status					
Never Smokers *n* (%)	7 (3.4%)	8 (4.4%)	6 (3.0%)	8 (4.2%)	0.87
Ever Smokers *n* (%)	196(96.6%)	173(95.6%)	195(97%)	182(95.8%)	
Educational Level					
Upper diploma *n* (%)	21(10.6%)	24(13.5%)	23(11.6%)	19(10.0%)	0.73
Diploma/Under diploma *n*(%)	182(89.4%)	157(86.5%)	178(88.4%)	171(90%)	

Values are expressed as mean (SD), median (IQR) or number (percentage). IQR, inter quartile range; BMI, body mass index; WC, waist circumference; HC, hip circumference; WHR, waist circumference to hip circumference ratio; FT4, free thyroxin level; TSH, thyroid stimulating hormone; TPO Ab, Thyroid peroxidase anti body

Table 2 demonstrates the prevalence of various types of thyroid dysfunction among participants with different AMH quartiles at initiation of the study; there was no significant difference between study groups in terms of thyroid dysfunction.

**Table 2:** Prevalence of thyroid disorders at the initiation of study according to the age-specific anti-Mullerian hormone (AMH) quartiles

Variables *N* (%)	1st quartile *n* = 203	2nd quartile *n* = 181	3rd quartile *n* = 201	4th quartile *n* = 190	*P*- value
TPO Ab+	41 (20.2%)	28(15.5%)	28(13.9%)	25(13.4%)	0.22
Subclinical Hypothyroidism	14 (6.9%)	8(4.4%)	4(2%)	8(4.3%)	0.12
Overt Hypothyroidism	7 (3.4%)	5 (2.8%)	5 (2.5%)	6 (3.2%)	0.94
Subclinical Hyperthyroidism	11 (5.4%)	11 (6.1%)	8 (4%)	8 (4.3%)	0.76
Overt Hyperthyroidism	3 (1.5%)	4 (2.2%)	7 (3.5%)	6 (3.2%)	0.56

Values are expressed as number (percent); TPO Ab+: Thyroid peroxidase antibody positivity

According to GEE analysis (Table 3), after adjustment for BMI, parity, smoking status, and educational level, there was a statistically significant annual decrease in mean changes of FT4 in all AMH quartiles; these decreases were [− 0.28(95%CI: -0.43, − 0.1); *p* < 0.001], [− 0.18, (95%CI: -0.34, − 0.01); *p* = 0.02], [− 0.25 (95%CI: -0.42, − 0.10); *p* = 0.001], [− 0.25 (95%CI: -0.41, − 0.09) pmol/L; *p* = 0.002] for 1st, 2nd, 3rd and 4th quartiles of AMH respectively. There was a statistically significant annual increase in TPO Ab in women of 1st quartile of AMH (10.66 ± 3.60 IU/ml), after adjustment for mentioned variables; the odds of TPO Ab positivity (TPO Ab+) in these women increased by 8% (95% CI: 0.06, 0.16) per year; these significant changes in TPO Ab was not observed in other quartiles of AMH (Table 3). There was no statistically significant difference in mean changes of TSH in all age-specific AMH quartiles (Table 3).

**Table 3:** Annual changes in various parameters according to age-specific Anti-Mullerian hormone (AMH) quartiles using generalized estimating equation (GEE)

Variables	1st quartile *n* = 203	2nd quartile *n* = 181	3rd quartile *n* = 201	4th quartile *n* = 190
FT4 (pmol/L)	−0.28 ± 0.07ª	−0.18 ± 0.07ª	−0.25 ± 0.07ª	−0.25 ± 0.07ª
TSH (mu/L)	0.58 ± 0.30	0.23 ± 0.32	0.05 ± 0.30	0.08 ± 0.31
TPO Ab (IU/ml)	10.66 ± 3.60ª	5.37 ± 3.94	6.49 ± 3.66	1.78 ± 3.68
TPO Ab+^b^	1.08ª (1.0006,1.16)	1.06(0.98,1.15)	0.95(0.87,1.04)	1.05(0.97,1.14)

ª*P* < 0.05 interaction form (follow-up year ×age-specific AMH quartile) (GEE); FT4, free thyroxin level; TSH, Thyroid stimulating hormone; TPO Ab, Thyroid peroxides anti body; TPO Ab+, Thyroid peroxidase antibody positivity, Data were adjusted for BMI, parity, smoking status, educational level

^b^Values are expressed as odds ratio and confidence interval

Based on GEE analysis, overall odds of TPO Ab+ in the first quartile of AMH was 2.05 fold higher than those of the fourth [95% CI:(1.31–3.74); *p* = 0.01], while the overall mean changes of TSH (*p*-value = 0.79), showed no statistically difference between these two quartiles, after adjustment for BMI, parity, smoking, and education level (Table 4). The interaction (follow-up year × AMH quartile status) in women in the first and the fourth quartiles of AMH was not statistically significant for any of the thyroid parameters (*p*-value > 0.05).

**Table 4:** Parameter estimates of GEE model in women in 1st quartile of AMH compared to those of women in the 4th quartile

Variable	Parameter	β coef.	SE	95% confidence interval	*P*-Value
TSH (mu/L)	*AMHQ1*	−0.48	1.8	(−4.16,3.19)	0.79
	*AMHQ4*	Reference			
	*Time*	0.33	0.44	(−0.53,1.21)	0.45
	*Time×AMHQ1*	0.63	0.62	(−0.58,1.85)	0.30
	*Time×AMHQ4*	Reference			
FT4 (pmol/L)	*AMHQ1*	−1.15	0.38	(−2.05,-0.12)	0.02ª
	*AMHQ4*	Reference			
	*Time*	0.38	−0.11	(−0.64,-0.12)	< 0.001ª
	*Time×AMHQ1* *Time×AMHQ4*	0.29	0.16	(−0.10,6.34)	0.06
		Reference			
TPO Ab+	*AMHQ1*	2.05	0.62	(1.31, 3.74)	0.01ª
	*AMHQ4*	Reference			
	*Time*	1.10	0.05	(1.004,1.22)	0.04ª
	*Time×AMHQ1*	0.93	0.06	(0.81,1.06)	0.28
	*Time×AMHQ4*	Reference			

*AMHQ1* AMH (Anti- mullerian hormone) in women of 1st quartile, *AMHQ4* AMH in women of 4th quartile, *BMI* body mass index, *FT4* free thyroxin level, *TSH* thyroid stimulating hormone, *TPO Ab* Thyroid peroxidase antibody, *TPO Ab+* Thyroid peroxides antibody positivity, × indicates interaction

**Fig. 2:**
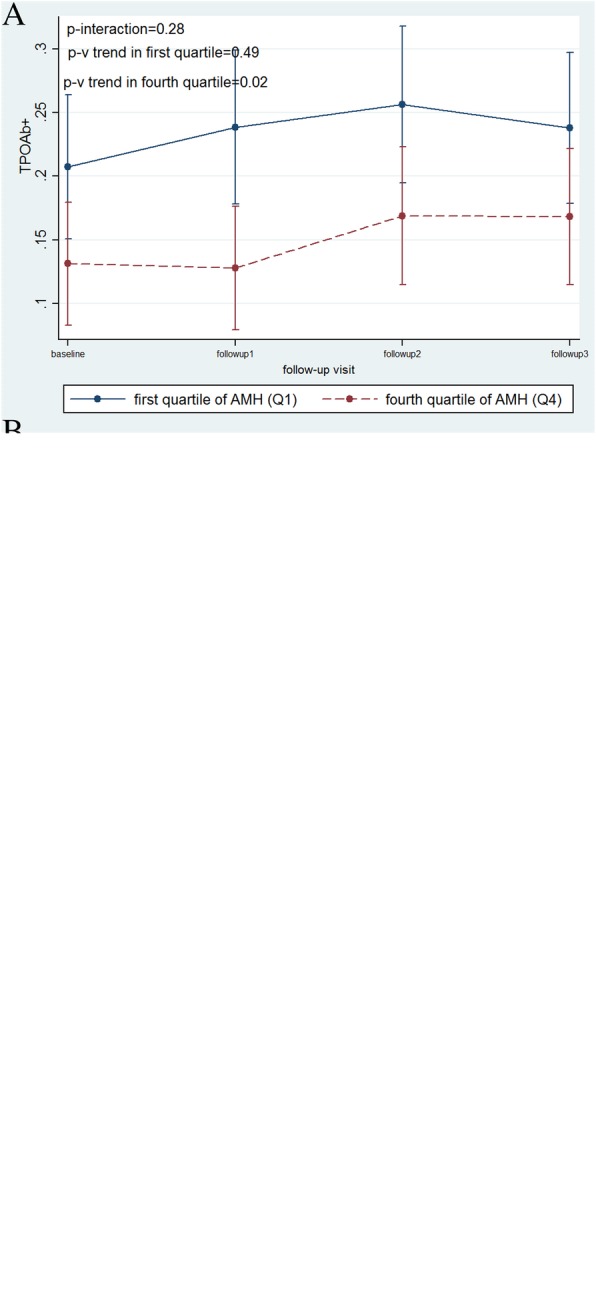
Mean of changes within follow-ups in the first and fourth quartiles of age specific AMH assuming the interaction between time and study group after adjusting for BMI, parity, smoking, and education level. **a** TPO Ab+ (Thyroid peroxidase antibody positivity), **b** FT4 (free thyroxin level), **c** TSH (thyroid stimulating hormone). First quartile of AMH (Q1), fourth quartile of AMH (Q4)

Figure 2 a, b, and c illustrate trends of TPO Ab, TSH and FT4 in women in the first and fourth quartiles of AMH, adjusted for BMI, parity, smoking, and education level. TSH had a significant increase in women in the first quartile (mean TSH = 2.4 mu/L in the first visit vs. 5.2 mu/L in the last visit, *P*_*trend*_ = 0.04); these values were 2.25 and 3.25 mu/L, *P*_*trend*_ < 0.001 for the first and last visits in the fourth quartile of AMH. However, mean changes of TSH in women in the first quartile were not significantly different from women in the fourth quartile (*P*_int*eraction*_ = 0.30)

Although FT4 showed a significant decrease in women in the fourth quartile (mean FT4 = 15.5 pmol/L in the first visit vs. 14.28 pmol/L the last visit,

< 0.001), FT4 of women in the first quartile showed no significantly different value overtime (*P*_*trend*_ = 0.14).

Interaction between follow-up years and AMH quartile demonstrate that as time progresses, changes in thyroid hormones of women in the first quartile of AMH do not differ or become significantly worse compared to those in the fourth quartile (Fig. 2).

There was no significant difference in incidence of subclinical hypothyroidism [HR:1.34 (95% CI:0.52,2.12)], overt hypothyroidism [HR:0.78(95% CI:0.34,1.80)] subclinical hyperthyroidism [HR:1.97(95% CI:0.59,6.63)], overt hyperthyroidism [HR:0.63(95% CI: 0.14,2.86)] and TPO Ab positivity [HR:1.49 (95% CI:0.73,3.03)] between those of the 1st versus those of the 4th quartile. Incidence rates of subclinical hypothyroidism in the 1st, 2nd, 3rd and 4th quartiles were [20(95% CI:10,27), 15(95% CI:10,23), 5 (95% CI:3,10); *p* = 0.002] and 14(95% CI: 10,21) per1000 person-years, respectively. This incidence in the 3rd quartile was significantly lower than in others, (*p* = 0.002).

## Discussion

The long term prospective population-based Tehran Thyroid Study provides a unique opportunity to investigate associations between overtime trend of thyroid functions and the ovarian reserve status. In the current study, we found that women with lower ovarian reserves had higher levels of TPO Ab at baseline; moreover a positive trend of this antibody was observed in these groups of women compared to those ones with better ovarian reserve status; there were more incidents of thyroid autoimmunity in the former group.

There are several studies with controversial results on association between hypothyroidism and thyroid autoimmunity with low ovarian reserve [9, 11, 21, 22]; however effects of low ovarian reserve on the overtime trend of thyroid function have not been investigated yet.

In agreement with our findings, Chen et al. (2017) in a cross-sectional study of 1044 infertile Chinese women demonstrated that idiopathic low ovarian reserve with lower serum concentration of AMH was associated with more frequent positive TPO Ab rather than thyroid function or Tg Ab positivity [21]. Saglam et al. in a case control study found that even after age adjustment, autoimmune thyroid disease(AITD) was independently associated with AMH, which was lower in AITD women than in controls [22]. In contrast, Polyzos et al. (2015) in a large retrospective cross-sectional analysis reported that thyroid autoimmunity was not associated with low ovarian reserve [9]. In an animal study, hypothyroidism in adult female rats was induced by an iodide deficient diet in combination with perchlorate supplementation for inhibition of iodide uptake by the thyroid. This condition in adult females negatively affects the ovarian follicular reserve and the size of the growing follicle population [11]. Moreover, some studies report that elevated serum concentration of TSH is associated with decreased serum level of AMH [4, 23, 24]. Weghofer et al. in a study of 225 infertile women reported that even after adjustment for thyroid autoimmunity and age, TSH < 3.0μIU/mL was associated with significantly better ovarian reserve and higher AMH than TSH ≥3.0μIU/mL [23]. In another study among 23 pairs of infertile and fertile women, it was found that both TSH levels and age were negatively correlated with AMH levels in infertile participants with standardized partial regression coefficient (β) of − 0.534 and − 0.361, respectively, but not in normal fertile ones [4]. This finding is in agreement with ours as we also found no association between serum concentration of TSH and ovarian reserve status among participants who were mostly fertile; since thyroid autoimmunity precedes thyroid dysfunction [25], it may need a longer follow up to cause significant elevation in TSH levels among women with thyroid autoimmunity.

The underlying pathophysiological mechanism of the association between thyroid autoimmunity and ovarian reserve status is not completely understood. In the Monteleone et al. study [26], for the first time the presence of thyroid antibodies in ovarian follicular fluid was demonstrated and a significantly lower oocyte fertilization and percentage of grade A embryos was found in infertile women with thyroid autoimmunity undergoing IVF compared to controls. Although this data are preliminary, but it may be assumed that TPO Ab that passes through the blood follicle barrier during follicular evolution may result in the destruction and damaging of growing follicles and oocytes via thyroid hormone receptors, on these cells [26]; a possible mechanism which needs to be confirmed by further comprehensive studies. Except for autoimmunity, thyroid dysfunction may influence the reproductive system via thyroid hormone receptors on the surface of oocytes [4], or through disruption of GnRH function due to increasing prolactin secretion [5].

Furthermore, in the present study, regardless of ovarian reserve status, we found that serum concentration of TSH had an incremental trend by age. In cross-sectional studies of individuals without thyroid disease, serum TSH concentrations increased with age [27–29], although this age-related TSH increase was not accompanied by a fall in free T4, suggesting an alteration in the TSH regulatory system [28, 30]. In the present study, the decline in FT4 among women with higher ovarian reserves is clinically unimportant. Additionally, some studies indicate hereditary and genetic effects on concentrations of free T4 and TSH, as well as on the negative feedback set point [31, 32].

We showed that the incidence of subclinical hypothyroidism in women of the 3rd AMH quartile was lower than those in other quartiles, even the 4th; this may partly be explained by the including of women with subclinical ovarian dysfunction in the 4th AMH quartile, who had higher AMH levels and regular menstrual cycles, but had polycystic morphology in sonography and subclinical anovulation [33, 34] and also higher rates of thyroid autoimmunity and hypothyroidism [35], despite our effort to recruit fertile women with regular menstrual cycles for the purpose of the present study.

The main strength of the present study is its methodology; it is the first study that has investigated the temporal trend of changes in thyroid hormones, TPO Ab and thyroid disorders among a general population of women with different levels of age specific AMH; in addition the follow up time was 12 years, which seems a sufficient duration for the purpose of the current study. We used age-specific AMH levels, instead of crude AMH levels because this method provides more accurate and precise assessment of ovarian reserve status [36, 37]. The intra-assay and inter-assay in our data is likely to be minimal because all thyroid assessments and AMH assays were performed in the same laboratory by the same expert.

This study had some limitations as well: 1) other methods for estimation of ovarian reserve including: FSH, antral follicular count, inhibin B were not assessed in the study; however AMH has been introduced to be the most reliable [15, 16, 36]. 2) Age specific AMH was identified according to a single AMH measurement at initiation of study and we did not evaluate ovarian reserve over time; however it has been shown that AMH level remains almost constant from one cycle to another and has a high interclass correlation coefficient, as a result of which only one measurement provides a reliable estimate of age specific level [38–40]. 3) Subjects were not assessed for the subclinical ovarian dysfunction, which may have affected the characteristics of women with higher ovarian reserves [41, 42]. 4) We did not measure Tg Ab in this study and thyroid autoimmunity was evaluated based only on serum TPO Ab measurement; however the presence of TPO Ab in serum is more frequent than Tg Ab [43]; furthermore a large population based study demonstrated a similar prevalence in both antibodies [44] and finally our study assessed only thyroid autoimmunity in relation to age specific AMH levels and autoimmunity in general was not assessed. Previous studies showed that ovarian reserve is closely linked to autoimmunity [22, 37]. In another study premature ovarian failure (POF) was significantly associated with adrenal cortex autoantibodies [45], which may be due to general autoimmunity that affects multiple organs and TPO Ab is a part of this process that has destructive effect on oocytes [26].

Large long-term studies are needed to evaluate both the causality between low ovarian reserve and appearance of thyroid autoimmunity, and the culprits that lead to the autoimmune process in women with limited ovarian reserve.

## Conclusion

In summary this study demonstrates that women with low ovarian reserves had both higher baseline and increasing levels of TPO Ab over a12 year follow. They had also a higher incidence of TPO Ab positivity over time. A prospective comprehensive study with several time points measurement of AMH and thyroid assessment is recommended to define the evolution of the association between ovarian reserve status and thyroid function.

## Abbreviations

AMH: Anti-mullerian hormone
BMI: Body Mass Index
DBP: Diastolic Blood Pressure
EIA: Enzyme Immunoassay
FT4: Free T4
GEE: Generalized Estimation Eq.
HC: Hip Circumference
IEMA: Immune Enzymometric Assay
IRES: Research Institute for Endocrine Sciences
PCOS: Poly Cystic Ovarian Syndrome
POF: Premature ovarian failure
SBP: Systolic Blood Pressure
TLGS: Tehran Lipid and Glucose Study
TPO Ab: Thyroid peroxidase antibody
TPO Ab+: TPO Ab positivity
TSH: Thyroid stimulating hormone
TTS: Tehran Thyroid Study
WC: Waist Circumference
WHR: Waist Circumference to Hip Circumference Ratio
